# The Effects of Park-Based Interventions on Health-Related Outcomes Among Youth: A Systematic Review

**DOI:** 10.1177/08901171221077812

**Published:** 2022-03-26

**Authors:** Deshira D. Wallace, Bing Han, Deborah A. Cohen, Kathryn P. Derose

**Affiliations:** 1 Department of Health Behavior, 41474 UNC Gillings School of Global Public Health, Chapel Hill, NC, USA; 2 166700 RAND Corporation, Santa Monica, CA, USA; 3 Department of Research and Evaluation, 6152 Kaiser Permanente Southern California, Pasadena, CA, USA; 4 Department of Health Promotion & Policy, School of Public Health and Health Sciences, 14707 University of Massachusetts Amherst, Amherst, MA, USA

**Keywords:** parks, interventions, physical activity, health outcomes, systematic review, youth

## Abstract

**Objective:**

The purpose of the study is to present a comprehensive systematic review of the effects of park-based interventions on health outcomes among youth, defined as children and adolescents.

**Data Source:**

Web of Science, MEDLINE, and Scopus databases searched through November 2020.

**Study Inclusion and Exclusion Criteria:**

Interventions conducted in publicly accessible parks that evaluated health outcomes (i.e., physical, mental, and emotional); focused on children and adolescents (up to 18 years old, or up to 22 years old for individuals with developmental needs); and was published in English, Spanish, and Chinese.

**Data Extraction:**

Two independent reviewers extracted data and assessed the quality of the 15 included studies using the Guide to Community Preventive Services tool.

**Data Synthesis:**

Descriptive summary of study characteristics and summarized methodological quality of the studies.

**Results:**

Twelve studies were person-based interventions involving the evaluation of health outcome changes in cohorts, and the remaining studies were park-based, focused on changing the park environment and observing changes in youth participation in parks. All identified interventions were positively associated with individual-level and park-level outcomes ranging from body weight, moderate-to-vigorous-intensity physical activity, park utilization, and health behavior knowledge.

**Conclusions:**

This systematic review demonstrated that parks as sites of interventions can provide an environment that promotes health and wellbeing for youth. Nevertheless, the number of relevant studies were limited, thus it is important to leverage and expand on existing knowledge of the utility of parks as sites of intervention to address health concerns at this critical juncture of the life course.

## Objective

Healthy behaviors engaged in during childhood are essential for school performance, learning, physical and mental health and development, as well as important in the prevention of chronic conditions through adulthood.^1,2^ One health behavior in particular, engagement in physical activity, contributes to improved physical fitness, bone health, cardiometabolic health, cognitive performance, and mental health such as reduced depressive symptoms and anxiety.^3^ Yet, evidence suggests that youth, defined by the World Health Organization as children and adolescents 0–19 years,^4,5^ are not meeting international physical activity guidelines, thereby contributing to poor health outcomes in childhood and later on in life.^1,6-8^ Globally 80% of adolescents aged 11 to 17 do not meet the physical activity recommendations of at least 60 minutes per day of moderate-to-vigorous-intensity physical activity (MVPA),^9^ and international studies of children from 3–6 years have found 21.2–35.6% of children do not engage in daily MVPA.^10,11^ A recent evidence summary of physical activity studies for children and adolescents 5–17 years found that of the 21 systematic reviews evaluated, each review was geared towards interventions that impacted physical activity, yet there were limited reviews that examined the association between physical health and mental health for youth.^9^ However, one meta-analysis that focused on the intersection of physical and mental health found a small but significant treatment effect of physical activity interventions on reduced depressive symptoms among youth 5–19 years old.^12^ A more recent meta-analysis evaluating physical activity intervention randomized controlled trials found a significant moderate overall effect on depressive symptom reduction.^13,14^

To promote physical activity as an important behavior, the environment in which youth engage in physical activity is critical. According to WHO guidelines, youth may be physically active by engaging in games, sports, recreation, transportation, physical education, planned exercise, and by playing,^15^ indicating a need to create local environments that facilitate increased movement in an equitable way across diverse settings to produce long-term health benefits for youth.^13^ A livable and sustainable built environment comprising urban green spaces, such as public parks, is tied to physical, mental, and emotional health and quality of life.^16^ In fact, according to the Task Force on Community Preventive Services, which makes recommendations on evidence-based interventions for disease prevention, creating or improving public places for physical activity was 1 of the recommended evidence-supported strategies for increasing physical activity.^17^ Public parks in particular address concerns as it relates to equity in access to spaces for play and physical activity unlike school-based programs or sports that are restricted to youth registered on a team or at a particular school.^18,19^ Park-based physical activity programs have the potential of being an ideal setting for child and adolescent chronic disease prevention and health promotion. Park systems, generally, provide free physical resources and facilities to be physically active and exposure to nature improves health and wellness.^20^ Given their accessibility and amenities, it is important to capitalize on parks as a point of intervention for child and adolescent health.

Further, public health studies show, and socio-ecological theoretical approaches that account for demographic characteristics, psychosocial factors, behavior variables, and socio-environmental factors suggest, that structural-level interventions (e.g., built environment and policies) can produce longer-lasting effects than studies that target the individual alone.^21-24^ Yet constructing structural interventions does not mean that all individuals will be affected equally. For instance, the effects of physical activity on health outcomes can be moderated by social positioning factors such as age, developmental stage, gender, socioeconomic status, and racialized and minoritized experiences.^25-27^ Thus, to be successful, programs that are situated in public parks requires not only an examination of access but equity in approaches to ensure that all youth can use parks’ resources to reach desired health outcomes.

The purpose of the study is to examine the role of publicly available parks in health-related outcomes of youth globally. This systematic review has two aims: to examine the state of the science on the physical, mental, and emotional health effects of interventions conducted at parks on youth; and determine topics that require additional areas of research.

## Methods

For this systematic review, we were interested in interventions conducted at parks or on parks to improve health-related outcomes among youth. This systematic review adopted and followed the reporting guidelines and criteria set in the Preferred Reporting Items for Systematic Review (PRISMA) statement.^28^ The protocol is registered with the International Prospective Register of Systematic Reviews (PROSPERO). More details on the protocol of the study were published elsewhere.

### Data Sources

Through an iterative process, the first author and the research librarian refined the search terms and databases. We read published literature reviews and key articles of interest to develop a list of key words and inclusion and exclusion criteria for the review. Key works and inclusion criteria were reviewed by co-authors and subject matter experts who have conducted park-based interventions and research. Once suggestions were agreed upon and integrated, we worked with a RAND research librarian to develop the key words, create a list of suggested databases, and iteratively create a finalized search strategy. The search was run in 3 databases, Web of Science, Pubmed/MEDLINE, and Scopus for peer-reviewed articles through November 2020. The search terms applied to each database were: parks, parklet, built environment, playfield, recreation center, green space, fitness zones AND physical activity, moderate to vigorous, MVPA, health*, physical health, mental health, sedentary, METs, metabolic equivalent task, intervention, AND RCT, randomized controlled trial, SOPARC, SOPLAY, observing play, recreation in communities, experiment, program, evalut*, direct observation AND child*, adolescent*, youth. We excluded terms: cattle, cows, elephant*, deer, boar, predator*, leopard*, national park, case report, comment, editorial, dissertation, thesis, blog, or newsletter. We reviewed the results of the literature search against known publications that fit our criteria to check for the search strategy’s sensitivity and made any necessary adjustments.

### Inclusion and Exclusion Criteria

The units exposed to the intervention (e.g., interventions focusing on the park, “park-based,” or focusing on individuals in the park, “person-based”) as well as the level at which the outcomes were measured (park-level or person level) were the important benchmarks for comparison. The inclusion criteria used to evaluate each case was: (a) peer-reviewed (published only), (b) published in English, Spanish, and Chinese as that enveloped the skills available in the research team, (c) evaluated health-related outcomes (physical, mental, emotional), (d) focused on children and adolescents (up to 18 years of age or up to 22 years of age for individuals with developmental needs), and (e) described an intervention conducted in a park accessible to the public. This final criterion was of particular importance for youth-focused park interventions, as park interventions needed to be conducted in publicly available parks or urban spaces, not schoolyards restricted to the school’s students.

Furthermore, we focused on rigorous study designs that used quantitative research methods. Study designs are interventions including randomized control trials, cluster-randomized trials, and quasi-experimental designs with or without comparison groups (i.e., one-arm pre-post study design).

Exclusion criteria were qualitative studies, abstracts, dissertation/thesis, blogs, newsletters, organization documents and government reports, book and book chapters, conference proceedings, case reports, and comments. In addition, we excluded studies broadly evaluating Public Open Spaces^29^ that assessed neighborhood-level characteristics such as sidewalks or those conducted in national parks. We also excluded intervention studies that were focused on sport team injury reduction if the study was not conducted in a park.

### Population of Interest, Exposure, and Outcome

This review included studies that intervened at the level of individuals (i.e., person-level), specifically interventions that compared an intervention group and comparison group or did a pre-post comparison after a health intervention at a park. Additionally, one-arm studies that evaluated health-related outcomes for the cohort at pretest and posttest were included. Studies that intervened at the park level were also included. Park-level interventions that compared park use and related health behaviors or health status before and after an environmental change, such as the addition of equipment, shade, or trails in the park were also included. There were no restrictions based on gender or geographic location of the study participants.

All health-related outcomes were eligible for this review. We extracted information from both the person-level and park-level. Person-level outcomes were those where the investigators intended to intervene directly on individuals by developing programs or cohort activities and thereby collected data from individuals before and after the intervention. Park-level outcomes were those in which the study authors intervened on the park, such as creating structural changes to the environment, to then observe aggregate behaviors and outcomes and/or follow individuals to collect relevant data.

### Data Extraction

**Figure 1.**
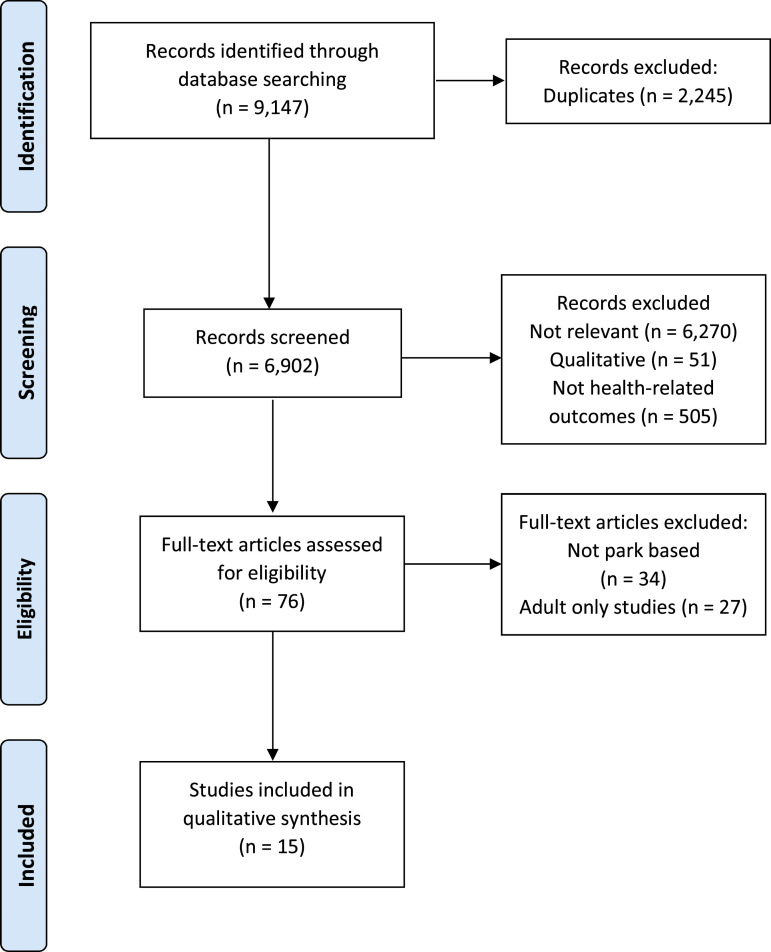
Preferred Reporting Items for Systematic Reviews and Meta-Analyses (Preferred Reporting Items for Systematic Review) diagram depicting review process.

All records were downloaded and deduplicated in EndNote (V8). The deduplicated list of records was imported into Covidence, an online, systematic review platform that allows for screening of records by multiple users (https://www.covidence.org/). For the title/abstract phase, 2 screeners trained on the inclusion criteria and experienced in systematic reviews screened all titles and abstracts for relevant records. Any conflicts on the inclusion or exclusion of records were discussed by the team. Then 2 screeners independently reviewed the full text of the articles against the inclusion and exclusion criteria and recorded reasons for exclusion in the Covidence system. Conflicts were reviewed by the lead author and senior authors to ensure quality assurance. The flow diagram depicting the process of study selection is found in Figure 1.

The final list of 15 articles underwent data extraction using the Community Guide’s *Guide to Community Preventive Services* tool.^30^ This tool contains 55 questions; however, we adapted some of the questions to account for the needs of this extraction for a total of 62 questions. The types of information extracted included (1) descriptive information (e.g., the purpose of the study, how the intervention was being delivered, geographic location, and study site); (2) the study population (e.g., eligibility criteria, demographic characteristics, and attrition details); (3) results (e.g., estimate, significance, and interpretation); and (4) study quality. We used 14 questions to assess study quality. Quality for each study was ultimately determined based on the level of detail provided by authors and study design type (e.g., RCT verses non-RCT) did not significantly bias study quality assessment. These questions were transcribed to an online survey platform, Qualtrics, which we used as a data entry form, and included both closed options and open-response options. Once trained on the tool, researchers extracted relevant information from the studies.

### Data Synthesis

We conducted a descriptive summary of study characteristics and evaluated and summarized the methodological quality of the included studies. We summarized interventions by exposure type and the primary outcomes reported by the study. No quantitative synthesis was performed; therefore, the coefficients of each reported health-related outcome were described at the study level. The quality of each eligible study was assessed using the validated Guide to Community Preventive Services.^30^ The key domains of the Guide to Community Preventive Services tool used to determine the quality of the studies are: description of the study, sampling type, measurement, analysis, interpretation of results, and other details. Within each domain are 2–6 questions to elicit information regarding contributions to poor study quality and therefore could limit the ability to interpret the results of the study. Each domain was averaged and then studies were classified as having “High,” “Medium,” or “Low” study quality based on the amount of information and characteristics of the study as described by the authors of each paper. Quality for all included studies was assessed and reviewed by the first and senior (last) authors.

## Results

We imported 9147 articles from our search, of which 2245 were removed as duplicates leaving 6902 articles for title and abstract screening. During the title/abstract screening we excluded 51 articles because the studies were qualitative, 505 articles because the authors did not examine a health-related outcome, and 6270 articles were excluded because they were not relevant to the research question and objectives of the study. Of the 76 remaining articles, 34 studies were not conducted at parks and 27 were only conducted with adults. The results of the adults-only studies are published elsewhere (masked for review). In total we found 15 studies that met our criteria. Twelve of the 15 studies were conducted in the United States (US), and several were specifically conducted in Florida. The 3 studies conducted outside of the US were in Australia, Denmark, and The Netherlands. All but one study was conducted in an urban center. Nine studies focused on low-income neighborhoods. Although 3 studies did not specify the mean age of participants but presented ranges or specified focusing on youth instead, for the studies that did report participant ages, the mean ages were between 8 and 13 years old. Details for each study is reported in Table 1.

**Table 1.** Demographic Characteristics for Youth-Focused Studies (n = 15).

Study	Country	Population Density	Neighborhood Socioeconomic Status	Target Population Description	Socioeconomic Status of Sample	Mean Ages (SD) in years
Bohn–Goldbaum, 2013	Australia	Urban	Low income	Children 5-12 residing in low-income neighborhoods in urban areas Race/ethnicity: NR	Low-income	not reported
Boonzajer Flaes, 2016	The Netherlands	Urban	Low income	Youth residing in low-income neighborhoods Race/ethnicity: NR	Low-income	not reported
Bush, 2007	USA (Texas)	Urban	Low income	Youth (6-12 years) Black: 61% Latino: 39%	Low-income	10.1 (1.3)
Colabianchi, 2011	USA (Ohio)	Urban	Low income	Youth (unspecified) Black students in schools: 66%	Not reported	not reported
D’Agostino, 2018	USA (Florida)	Urban	Not specified	Youth (6-15 years) with severe obesity Black: 51% Latino: 49%	Low and middle income	9.4 (nr)
D’Agostino, 2018	USA (Florida)	Urban	Low income	Youth (6-15 years) Black: 49% Latino: 51%	Low and middle income	9.1 (nr)
Fair, 2017	USA (South Carolina)	Not reported	Not specified	Youth (up to 18 years) White: 90.3% Non-white: 9.7%	Middle and high income	8.2 (3.1)
Frazier, 2015	USA (Unspecified)	Urban	Low income	Youth (12-14 years) Black: 100%	Low-income	Group 1: 12.9 (1.0) Group 2: 12.8 (1.2) Group 3: 13.4 (.5)
George, 2016	USA (California)	Urban	Low income	Youth (9-14) who are overweight/obese *Intervention:* Black: 29% Latino: 59% White: 13%	Low-income	Intervention: 11.9 (1.5) Control: 11.2 (1.6)
				*Control:* Black: 76% Latino: 21% White: 4%		
Haney, 2014	USA (Florida)	Urban	Low income	Youth (up to 22 years) with developmental disabilities Black: 23% Latino: 65% White: 12%	Not reported	13.7 (4.7)
Messiah, 2017	USA (Florida)	Urban	All SES	Youth (6-14 years) Black: 44% Latino: 51% White: 3% Not specified: 2%	Low and middle income	9.1 (2.11)
Messiah, 2018	USA (Florida)	Urban	All SES	Youth (6-14 years) Black: 47% Latino: 50% White: 3%	Low and middle income	9.1 (2.11)
Messiah, 2018	USA (Florida)	Urban	All SES	Youth (6-14 years) Black: 47% Latino: 50% White: 3%	Low and middle income	9.1 (2.11)
Messiah, 2019	USA (Florida)	Urban	All SES	Youth with intellectual disabilities (6-22 years) Black: 20% Latino: 70% White: 10%	Low and middle income	14.1 (4.4)
Pawlowski, 2019	Denmark	Urban	Low income	Youth (fifth grade) Race/ethnicity: NR	Low and middle income	10.8 (.6)

nr = not reported.

### Person-Based Studies

The most common studies were person-based studies (12 of 15), specifically that the intervention was person-based, directed to individuals and involved the examination of health changes among specific cohorts (Table 2). The majority were one-arm, pre-post studies. Samples sizes ranged from 39 ^31^ to 2464 children,^20^ with the interventions lasting between 4 weeks and 10 months. Most intervention studies were conducted in collaboration with school partners.

**Table 2.** Intervention and Outcome Characteristics of Youth-Focused Studies (n=15).

Study	Sample Size, Description	Intervention Description	Study Design	Primary Park-Level Outcomes		Primary Person-Level Outcomes
Person-Based interventions						
Bush, 2007^a^	120 children between 6-12 years-old children (recruited from community centers)	Community-based organization delivered Project KidFIT consisting of nutrition courses and outdoor physical activity to support a healthy lifestyle	6 weeks: 1-arm; pre-post			Body weight** • [Clinical exam] Δ= .33 kg, SE = .24
						Strength & flexibility** • [Clinical exam] Non-overwgt: μ =7.0, SE=1.5 Overwgt: μ = 7.2, SE = 1.0
						Nutrition knowledge** • [Self-report] μ=2.5 points, SE=n/r
D’Agostino, 2018a^a^	176 children between 6-14 years old who are severely obese	Fit2Play, a multisite park-based afterschool program using the sports, play and active recreation for kids (SPARK) curriculum focused on 40-minutes of outdoor physical activity delivered by park coaches with oversight from health and wellness specialists	10 months: 1-arm; pre-post			Blood pressure** • [Clinical exam] Adj IRR Sys BP: .95 (95CI: .92, .97) Dias BP: .94 (95CI: .91, .97)
						PACER score (laps)** • [Fitness exam] Adj IRR: 1.22 (95CI: 1.10, 1.32)
						BMI** • [Clinical exam] Adj. IRR: .98 (95CI: .96, .99)
D’Agostino, 2018b^a^	2250 children between 6-14 years old	Fit2Play, a multisite park-based afterschool program comprised of outdoor physical activity and nutrition and wellness counseling	10 months: 1-arm; pre-post			BMI** • [Clinical exam] Adj IRR Black: .85 (95CI: .84, .87) Latino: .86 (95CI: .83, .88)
						Skinfold thickness** • [Clinical exam] Adj IRR Black: .85 (95CI: .83, .88) Latino: .85 (95CI: .83, .89)
						Blood pressure** • [Clinical exam] Adj IRR Sys BP: Black: .90 (95CI: .88, .95) Latino: .98 (95CI: .95, 1.01)
						400m run time** •[Fitness exam] Adj IRR Black: .94 (95CI: .92, .95) Latino: 1.06 (95CI: 1.04, 1.07)
Fair, 2017	746 families (parent and child pairs)	Park hop intervention that incentivized park usage through a scavenger hunt initiative	4 weeks: 1-arm; pre-post		Physical activity • [SOPARC] Or: 1.61 (95CI: 1.19-2.20)	Parks visited (1 month)** • [Self-report] t=1.97, P = .05
						Park discovery** • [Self-report] μ = 4.6, SD = 3.7
Frazier, 2015^a^	46 children aged 12-14 years old	Park program for middle school youth that leveraged the strengths and capacities into recreational activities and fostered resilience among youth and families	10 weeks: 3-arm study comparing a behavioral intervention in 3 different neighborhood parks; non-random			Staff-reported problem behaviors** •[Self-report] ES (postest): .46 ES (followup): .88
						Staff-reported social skills** • [Self-report] ES (posttest): −.72 ES (followup): −.53
George, 2016^a^	155 children aged 9-14 who are overweight or obese	Health lifestyle fitness camp was comprised of nutrition education and 3-hours of daily physical activity	6 weeks: 2-arms; non-random; pre-post			Weight reduction** • [Clinical exam] μ = −1.30 kg, SE=.14
						Waist circumference** • [Clinical exam] μ = −3.19 cm, SE = .33
						Waist to height ratio** • [Clinical exam] μ= −.032, SE=.002
Haney, 2014^a^	52 children aged 5-22 with developmental disabilities	Fit2Play program, aimed to increase outdoor physical activity, adjusted for children with disabilities	10 months: 1-arm; pre-post			BMI •[Clinical exam] ns
						Sit-ups** • [Fitness exam] ES: .21, P< .01
						Push-ups** • [Fitness exam] ES: .46, P<.0.046
						Health/wellness knowledge** • [Self-report] ES = 1.10, P < .01
Messiah, 2017^a^	2464 children aged 6-9 years and 10-14 years	Fit2Play, a multisite park-based afterschool providing children with 40-minutes a day of outdoor physical activity, including playing sports (SPARK) as well as a nutrition curriculum (EmpowerMe4Life)	10 months: 1-arm; pre-post			BMI** • [Clinical exam] μ = .7, SD=0.3
						Sit-ups** • [Fitness exam] μ = 27.7, SD = 13.3
						Systolic/Diastolic Blood Pressure** • [Clinical exam] μ = 69.5, SD = 24.6
Messiah, 2018a^a^	2115 children aged 6-9 and 10-14 years	Fit2Play, a multisite park-based afterschool providing children with 40-minutes a day of outdoor physical activity, including playing sports (SPARK) as well as a nutrition curriculum (EmpowerMe4Life)	10 months: 1-arm; pre-post			PACER score (laps)** • [Fitness exam] Adj IRR
						*Girls* Yr1: 1.10 (95CI:1.01, 1.19) Yr2: 1.23 (95CI:1.04, 1.45) Yr3: 1.34 (95CI:1.06, 1.70)
						*Boys* Yr1: 1.14 (95CI:1.06, 1.24) Yr2: 1.29 (95CI:1.12, 1.47) Yr3: 1.27 (95CI:1.02, 1.60)
						400-M run time** • [Fitness exam]
						*Girls* Yr1: .92 (95CI:0.87, .97) Yr2: .86 (95CI:0.77, .96) Yr3: .65 (95CI:0.92, .98)
						*Boys* Yr1: .91 (95CI:0.86, .96) Yr2: .91 (95CI:0.82, 1.01) Yr3: .83 (95CI:0.70, .98)
						Push-ups** • [Fitness exam]
						*Girls* Yr1: 1.14 (95CI: 1.06, 1.22) Yr2: 1.16 (95CI: 1.01, 1.32) Yr3: 1.09 (95CI: .88, 1.35)
						*Boys* Yr1: 1.10 (95CI: 1.04, 1.17) Yr2: 1.02 (95CI: .91, 1.15) Yr3: .99 (95CI: .83, 1.17)
Messiah, 2018b^a^	2115 children aged 6-9 and 10-14 years	Fit2Play, a multisite park-based afterschool providing children with 40-minutes a day of outdoor physical activity, including playing sports (SPARK) as well as a nutrition curriculum (EmpowerMe4Life)	10 months: 1-arm; pre-post			Skinfold thickness** • [Clinical exam] μ =-8 mm; P < .0.05
						Diastolic blood pressure** • [Clinical exam] Adj IRR Yr1: −2.43 (95CI: −3.65, −1.21) Yr2: −6.69 (95CI: −9.29, −4.09) Yr3: −5.87 (95CI: −9.46, −1.31)
						PACER score (laps)** • [Fitness exam] Adj IRR Yr1: 2.22 (95CI: 1.48, 2.95) Yr2: 2.37 (95CI: .79, 3.96) Yr3: 5.61 (95CI: 2.99, 8.24)
Messiah, 2019^a^	297 youth aged 6-22 years living with an intellectual disability	Fit2Play, a multisite park-based afterschool program focused on health (60 minutes of daily physical activity) and wellness (SPARK) among youth with intellectual and/or developmental disabilities who are primarily from ethnic minority backgrounds	10-months: 1-arm; pre-post, and 2 years post participation			Skinfold thickness** • [Clinical exam] Adj IRR Yr1: 1.02 (95CI: .99-1.05) Yr2: .96 (95CI: .93-.99)
							Systolic Blood Pressure** • [Clinical exam] Adj IRR Yr1: .94 (95CI: .92-.96) Yr2: .90 (95CI: .87-.93)
							Diastolic Blood Pressure** • [Clinical exam] Yr1: .87 (95CI: .85-.90) Yr2: .81 (95CI: .78-.84)
							Pacer score, laps** • [Cardiovascular fitness] Yr1: 1.36 (95CI: 1.28-1.45) Yr2: 1.57 (95CI: 1.44-1.70)
Pawlowski, 2019		39 fifth-grade children; 5 playable installations in public open spaces	Used participatory approaches with fifth-grade children to create tailored, playable installations in public open spaces	6 months: 1 arm, pre-post			Light physical activity • [Accelerometer] minutes Baseline: 22.8 Follow-up: 19.8
										MVPA • [Accelerometer] minutes Baseline: 23.4 Follow-up: 0.5
				Park-Based interventions						
				Bohn-Goldbaum, 2013	2 parks; 1 renovated, 1 unrenovated park; targeted children aged 5-12 years	Natural experiment comparing renovated parks with updated playgrounds and unrenovated parks to study park usage and physical activity of children and parents	2 weeks: 2-arms; non-random; pre-post	Resource-utilization • [SOPARC] ns		Physical activity • [Parent-reported] ns
									MVPA** (girls) • [SOPARC] Pre: 1.14 (2.37) Post: .24 (.44) (P =.04)	
									MVPA (boys) • [SOPARC] ns	
				Boonzajer Flaes, 2016^a^	20 parks; 10 renovated, 10 unrenovated parks; targeted children in low-income neighborhoods	Test if renovated public playgrounds (krajicek) in low-income neighborhoods stimulate daily physical activity and higher usage than in unrenovated parks	3 months: 2-arms; non-random; cross-sectional		No. Park visitors** • [SOPLAY] Or: 5.2 (95CI: 2.0, 13.7)	
									No. children in parks** (boys) • [SOPLAY] Or: 1.8 (95CI: 1.1, 3.1)	
									Energy expenditure** • [SOPLAY] b: .006 (95CI: .004, .008)	
				Colabianchi, 2011^a^	20 parks; 10 renovated; 10 unrenovated parks; targeted elementary aged children	Afterschool park usage and physical activity intensity observation between renovated (play features, shade, benches) and unrenovated parks	4 weeks: 2-arms; non-random			Overall safety and MVPA** (boys) • [EARPS] b: −.48 (95CI: −.88, −.07)
										Presence of benches & MVPA** (boys) • [SOPLAY] b: .12 (95CI: .01, .24)
										MVPA & shade** (boys) • [SOPLAY] b: .07 (95CI: .01, .12)

Note. SOPARC = System for Observing Play and Recreation in Communities. SOPLAY = System for Observing Play and Leisure Activity in Youth. EARPS = Environmental Assessment of Public Recreation Spaces. BMI = body mass index. MVPA = moderate-to-vigorous-intensity physical activity. n/r/= not reported; ns = not significant. μ = mean. ES = effect size. b = beta. CI = confidence interval. IRR = incident rate ratio.

**Significant difference between intervention group(s) and comparison group(s).

^a^Intervention was effective at changing targeted health outcome.

An example of a cohort study conducted with school partners at a park is Fit2Play, used in 7 studies.^20,32-37^ Fit2Play is a structured multisite afterschool program housed in urban county park systems.^20^ The program was conducted in 34 Miami Dade county parks, 2:00pm-6:00pm daily between the 2010–2011 and 2014–2015 school years aimed at improving the wellbeing of Hispanic/Latino and non-Latino Black children. The daily program comprised 60 minutes of physical activity incorporating multiple sports. The sport-based curriculum came about from the Sports, Play, and Active Recreation for Kids (SPARK) program, which is a play and evidence-based outcome oriented structured active recreation program for children with the purpose of developing and improving motor skills, movement knowledge, and social and personal skills.^38^ Participants had 20-30 minutes of nutritional education up to 2 times a week using the EmpowerMe4Life curriculum, which was developed at a fifth-grade reading level and was based on the American Heart Association’s recommendations for heart health.^39^ All participants completed baseline measures at the beginning of the school year (August/September) and post-test measure at the end of the school year (May/June). Measures collected were demographics, clinical measurements such as height, weight, waist circumference and skinfold thickness, and blood pressure (systolic and diastolic) by a trained team. Additional measures collected were physical fitness measures using a standardized and validated testing protocol for children and adolescents (flexibility, muscular endurance, sit-ups, push-ups, and aerobic fitness).

Results from Fit2Play studies found significant reductions among Black and Latino children in body mass index (BMI),^20,33,34^ skinfold thickness,^33,35^ and reductions in systolic and/or diastolic blood pressure.^20,33,34,36^ Physical fitness measures such as run time, sit-ups, and laps also improved from baseline to post-test. As the program worked well for children overall, additional studies examined the effects of the Fit2Play intervention on specific subgroups, in particular, youth with intellectual and developmental disabilities, who may be at greater risk of overweight and obesity. In the Haney et al^32^ study, the authors found that participation in the program led to significant improvements in fitness test measures as well as health and wellness knowledge for both normal weight and overweight or obese children/adolescents aged 6–22 years with intellectual and/or developmental disabilities. In another study Messiah et al^36^ examined the program for children and adolescents with developmental disabilities aged 6–22 years and found significant improvements in skinfold thickness and blood pressure, as well as fitness scores.

One example of person-based interventions included integrating physical activity and nutritional counseling,^40,41^ which resulted in significant weight reduction, increases in nutritional knowledge, and playing outdoor games to promote physical activity,^42^ thereby significantly increasing physical activity and park utilization. Another person-based intervention improved emotional wellbeing (i.e., equilibrium between resources and challenges)^43^ and proxy-reported social skills.^44^ Not all interventions produced significant results, such as one study that used participatory approaches to improve open spaces to increase physical activity for younger children.^31^ Relatedly, all but two person-based studies were effective at changing a health-related outcome among children.^31,42^ Five studies conducted follow-ups after post-tests to assess longer-term intervention effects.^33-35,37,41^ Studies using the Fit2Play intervention reported that after 2 years, improvements in cardiovascular health were associated with parks in less segregated areas, indicating a need to examine the social contexts where parks are located.^33-35^

### Park-Based Studies

There were three park-based interventions that fit our inclusion criteria. These studies focused on park renovations and the observation of physical activity, often in the form of “playing” among youth who attended these parks. The interventions were geared towards families with low income. The observation time ranged from 2 weeks to 3 months, and the observations were conducted using validated tools such as the System for Observing Play and Recreation in Communities (SOPARC)^45^ or the System for Observing Play and Leisure Activity in Youth (SOPLAY).^46^ The Bohn–Goldbaum^47^ study, conducted in Australia, was a natural experiment comparing renovated and unrenovated parks to examine if renovation led to increased usage and physical activity by youth. The authors found a statistically significant decrease in MVPA engagement among girls from pre to post intervention in the renovated park, as measured in MVPA engagement in a 2-hour observation period (SOPARC MVPA Δ: .90, *P* = .04). MVPA among boys increased in the renovated park, but the change was not significant. The authors posit that the decline in MVPA among girls may have been due to the way the park was renovated, specifically dispersed play equipment and the addition of amenities (picnic tables and open spaces) that may have been associated with sedentary behavior. Boonzajer Flaes et al^48^ conducted a study in The Netherlands that compared 10 unrenovated and 10 renovated parks (e.g., loose equipment, updated permanent equipment, fenced areas) in low-income neighborhoods, using SOPLAY, and reported increased number of park visitors, and increased physical activity (i.e., energy expenditure) among children and adolescents in the renovated parks over the 3-month observation period. However, the authors noted that there was a significantly higher number of boys participating in physical activity in the renovated parks than girls. Similarly, Colabianchi et al^49^ examined 10 renovated (i.e., new playground and safety equipment and park improvements) and 10 unrenovated parks in Cleveland, Ohio for afterschool use and physical activity for 4 weeks using SOPLAY. Observers reported that the number of play features was positively associated with utilization of the renovated parks. However, more play features were not significantly associated with increased MVPA levels in children. In addition, the presence of shade and benches were associated with positive utilization and with increased proportion of MVPA for boys, but not for girls. Overall, park-based interventions were most effective at increasing utilization at parks more so than observed changes in physical activity, except for Boonzajer Flaes,^48^ who found higher energy expenditures in children playing in renovated parks compared to unrenovated parks. Lastly, only Boonzajer Flaes^48^ reported intervention effects after the post-test period.

### Study Quality

Study quality was assessed using 5 domains: description of the study, description of sampling, measurement and scale information, data analysis information and appropriateness, and interpretation of results. Most studies (12 of 15) had an average of medium to high study quality. Data analysis was noted as medium to high quality due to most authors specifying and providing a rationale for their statistical analyses, which controlled for designed effects, repeated measures, and noted limitations to the analytical approach. The 3 studies that were assessed as low quality were due to poor descriptions of sampling design (sampling frame, screening criteria, use of probability sampling, and selection bias addressed), not specifying reliability or validity information of scales used, and/or a limited interpretation of the results (Table 3).

**Table 3.** Study Quality Assessment: Risk of Bias.

First Author, year	Description of Study	Sampling	Measurement	Data Analysis	Interpretation of Results	Average
Bohn-Goldbaum, 2013	Medium	Low	Medium	Medium	High	Medium
Boonzajer Flaes, 2016	High	Low	Medium	High	Medium	High
Bush, 2007	High	Low	Low	Medium	Low	Low
Colabianchi, 2011	Low	Low	High	Medium	Low	Low
D'Agostino, 2018	High	High	Low	High	Low	Medium
D'Agostino, 2018	High	High	High	High	High	High
Fair, 2017	Medium	Low	Low	Medium	Low	Low
Frazier, 2015	High	Low	High	Medium	Medium	Medium
George, 2016	High	Medium	Medium	Low	High	Medium
Haney, 2014	High	High	High	Medium	High	High
Messiah, 2017	High	Medium	High	High	High	High
Messiah, 2018	High	Medium	High	High	Medium	High
Messiah, 2018	High	Medium	High	High	Medium	High
Messiah, 2019	High	High	High	High	Medium	High
Pawlowski, 2019	High	Low	Medium	Medium	Medium	Medium

## Conclusion

In this systematic review our goal was to examine the state of the science as it relates to interventions conducted at public parks targeting youth to improve health-related outcomes. Public parks as sites of public health interventions are thought to support positive health behaviors and outcomes among youth because they exist in most communities, allow for time in nature, and are accessible to youth of diverse social and economic positions.^50,51^ We found that interventions in public parks, either as group activities or structural changes, can result in improvements in health-related outcomes for children and adolescents; thus, parks are critical spaces to promote population health to improve health and wellbeing. Most studies we evaluated focused on physical health, using measures of physical activity or fitness, or clinical measures such as calculated BMI, waist circumference or blood pressure, as compared to the 1 study focused on emotional health outcomes.^44^ However, given the limited evidence on mental and emotional health outcomes of park-based interventions among youth, additional research is needed to elucidate the role of park use and mental and emotional health among youth.

For youth, person-based studies were the most common intervention type. These interventions ranged from large, multi-year interventions with over 2000 participants, to smaller studies involving fewer than 50 participants. The 3 park-based intervention studies all compared renovated and unrenovated parks. Despite the limited number of park-based interventions identified, all 3 were robust in terms of the measurement of health outcomes with the use of validated instruments (e.g., SOPLAY). This predominance of person-based interventions targeting youth contrasts with a recently published article evaluating park-based intervention studies for adults and/or the general public, where park-based interventions dominated (masked for review). Of 27 interventions, 20 were park-based and 7 were person-based. One reason for the difference between published studies on park-based interventions among youth vs adults may be due to how person-based studies were structured for youth. Although conducted at parks, study teams often collaborated with schools to recruit youth participants. Schools are often seen as an ideal setting for health promotion interventions aimed at children and adolescents due to the efficiency and effectiveness of reaching the majority of the target population.^52^

Although the studies produced generally consistent results on several physical health outcomes, youth is a broad construct encompassing children and adolescents; therefore, accounting for any differences in their development is important to consider for various outcomes. For example, developmental periods affect differences in park use. In an observational study conducted in 20 parks New York City, Marquet and colleagues^53^ found that the average 5–10-year-olds had higher energy expenditure (metabolic equivalent tasks [METs]) than the average teenager. Relatedly, other demographic characteristics require explicit examination, for instance, Boonzajer Flaes and colleagues noted that there were differences in park usage by gender, with renovated parks in socioeconomically disinvested neighborhoods associated with increases in park use among boys but not girls.^48^ Further, Huang et al^50^ found that neighborhood characteristics were associated with park use among children 5–10 years old in socioeconomically disinvested neighborhoods. For example, for all children, the level of neighborhood socioeconomic disinvestment and crime was negatively associated with MVPA at neighborhood parks.^50^ These indicate that there is a need to account for differences in outcomes by subgroups and tailor interventions as well as develop policies to address neighborhood disinvestment to ensure equity in health outcomes.

It is important to note that the use of parks as intervention sites also affects non-health related outcomes among youth. For example, D’Agostino et al^54,55^ examined the changes in youth arrests after engagement in a park-based mental health promotion program (Fit2Lead) in youth.^56^ Improving park environments, by which youth, especially youth of color or those who live in economically precarious positions, do not feel criminalized and overly surveilled, can in turn increase youth engagement in neighborhood and city parks and public spaces.^57^ Research that continues to examine the role of parks as not only sites to change health behaviors, but environments to modify social conditions that are ultimately linked to health, are needed.

### Limitations

Park-based interventions consistently showed positive effects on health outcomes among youth across global settings. However, there were several limitations in the current review. Although the intention was to examine interventions conducted at parks globally, most studies were conducted in a limited number of countries (Australia, Denmark, The Netherlands, US). Additional research is needed to examine the role of parks for youth health and wellbeing in developing countries, specifically to better understand the practicality and utility of such intervention types in different settings. Similarly, all but one study was conducted in urban centers, thus gaps in knowledge exist regarding the implementation and effectiveness of public park interventions in settings with different levels of urbanicity. Of note, this review excluded studies that focused on organized sports, did not have a health-related outcome, were not conducted in a park, or did not comprise an intervention. In relation to organized sports, or sports leagues, there were studies that focused on the health of registered youth athletes; however, they were excluded because they focused on outcomes specific to their sport (e.g., endurance and injury prevention) and were not always conducted in parks, as defined by this review. Recently published reviews on youth participation in organized sports participation found positive association between sport and injury prevention,^58^ mental health,^59^ and pediatric obesity.^60^ Additionally, most studies focused on physical health outcomes; therefore, additional public park intervention studies are needed to examine how engagement in park activities are associated with a variety of mental and emotional health outcomes. Finally, seven studies were based on the Fit2Play intervention, which provides substantial evaluation of the Fit2Play intervention; however, it indicates a need for more diverse interventions in other contexts in the US and globally.

### Implications

This systematic review demonstrated that as sites of interventions, parks can provide an environment that promotes health and wellbeing for children and adolescents. Most interventions examined for this review were person-based, which showed promising results in changing health-related behaviors. Although fewer studies were park-based, these also demonstrated that improvements in the structure of the park led to greater utilization and more movement (e.g., MVPA) among youth, all of which can be linked to improvements in health. As health during early points of development affect mental, emotional, and physical health trajectories later in adulthood, it is important to leverage and expand on existing knowledge of the utility of parks as sites of intervention to address health concerns over the lifespan.

**So What?**

**What Is Already Known on This Subject?**

Interventions conducted at public parks are effective sites to promote health of youth.

**What Does This Article Add?**

This review examined interventions conducted in public parks that focused on youth to address physical, mental, and emotional health outcomes, and found that parks did improve measured health outcomes in youth. Most interventions were cohort-studies and fewer interventions involved environmental changes to the park to evaluate if and how renovations led to changes in park usage and health outcomes.

**What Are the Implications for Health Promotion Practice or Research?**

This review found that interventions conducted at parks have been shown to improve the health and wellbeing of children and adolescents. However, most interventions focused on physical health outcomes, rather than youth’s mental or emotional health. Therefore, future interventions are needed that address children and adolescents’ emotional and mental health, as well as physical health more holistically.

## Appendix

### List of Abbreviations

BMI Body: Mass Index
METs: Metabolic equivalent tasks
MVPA: Moderate-to-vigorous physical activity
PRISMA: Preferred Reporting Items for Systematic Review
RCT: Randomized controlled trial
SOPARC: Systems for Observing Play and Recreation in Communities
SOPLAY: System for Observing Play and Leisure Activity in Youth
SPARK: Sports, Play, and Active Recreation for Kids
